# Adding artesunate to sulphadoxine-pyrimethamine greatly improves the treatment efficacy in children with uncomplicated falciparum malaria on the coast of Benin, West Africa

**DOI:** 10.1186/1475-2875-6-170

**Published:** 2007-12-21

**Authors:** Alain Nahum, Annette Erhart, Dorothée Gazard, Carine Agbowai, Chantal Van Overmeir, Harry van Loen, Joris Menten, Martin Akogbeto, Marc Coosemans, Achille Massougbodji, Umberto D'Alessandro

**Affiliations:** 1 Laboratoire de Parasitologie, Centre de Recherches Entomologique de Cotonou, Cotonou, Bénin; 2 Department of Parasitology, Prince Leopold Institute of Tropical Medicine, Antwerp, Belgium; 3 Laboratoire de Parasitologie de la Faculté des Sciences de la Santé, Université Nationale du Bénin, Cotonou, Bénin

## Abstract

**Background:**

Benin has recently shifted its national antimalarial drug policy from monotherapies to combinations containing artemisinin derivatives. When this decision was taken, the available information on alternatives to chloroquine and sulphadoxine-pyrimethamine, the first- and second-line treatment, was sparse.

**Methods:**

In 2003 – 2005, before the drug policy change, a randomized, open-label, clinical trial was carried out on the efficacy of chloroquine, and sulphadoxine-pyrimethamine alone or combined with artesunate, with the aim of providing policy makers with the information needed to formulate a new antimalarial drug policy. Children between six and 59 months of age, with uncomplicated malaria and living in the lagoon costal area in southern Benin, were randomly allocated to one of the three study arms and followed up for 28 days.

**Results:**

Treatment failure (PCR corrected) was significantly lower in the artesunate + sulphadoxine-pyrimethamine group (4/77, 5.3%) than in chloroquine group(51/71, 71.8%) or the sulphadoxine-pyrimethamine alone group (30/70, 44.1%) (p < 0.001). Despite high sulphadoxine-pyrimethamine failure, its combination with artesunate greatly improved treatment efficacy.

**Conclusion:**

In Benin, artesunate + sulphadoxine-pyrimethamine is efficacious and could be used when the recommended artemisinin-based combinations (artemether-lumefantrine and amodiaquine-artesunate) are not available. However, because sulphadoxine-pyrimethamine is also used in pregnant women as intermittent preventive treatment, its combination with artesunate should not be widely employed in malaria patients as this may compromise the efficacy of intermittent preventive treatment.

## Background

In Benin, early malaria case management, both at health facility and community level, is the backbone of the national malaria control strategy [1]. High chloroquine (CQ) resistance, the first-line treatment for many years, has lead to a review of the national antimalarial policy in 2004 [1-3]. Two artemisinin-based combination treatments (ACTs) were recommended for uncomplicated falciparum malaria, namely artemether-lumefantrine (AL) and amodiaquine-artesunate (AQ-AS). Unfortunately, the implementation of the new national antimalarial drug policy currently meets several obstacles. Despite their availability in the pharmacies of the economic capital, Cotonou, and of other major cities, these ACTs are still not available in the public health facilities, and even less at community level. Thus, the large majority of the population does not have access to this newly recommended treatment, mainly because of its cost. Consequently, malaria patients continue to be treated with CQ, despite its low efficacy.

In Benin, CQ resistance was first reported in 1986 [4-6] and, in 1998, it was estimated at 23.8% in the south and 21.4% in the north, at day 14 after treatment (A. Massougbodji, personal communication) according to the 1996 WHO guidelines [7]. CQ resistance was higher (35.2%) when the follow-up was extended to 28 days after treatment [3,8].

Sulphadoxine-pyrimethamine (SP) efficacy was assessed in 2002 in five different sites with varying intensity of malaria transmission [3]. It had a patchy distribution with an overall treatment failure at day 28 of 22.8%, but ranging from 3.3% to 45.9%. However, it should be noted that none of these estimates was corrected by genotyping and the true treatment failure, excluding new infections, is probably lower. SP is currently used for intermittent preventive treatment (IPT) in pregnant women and there are plans to use it as IPT in infants [1,9]. No data on alternative treatments to CQ and SP were available in Benin at the time when the national drug policy was revised. A randomized clinical trial was carried out on the efficacy of the first- and second-line treatment, CQ and SP-alone, and SP associated with artesunate (SP-AS), in children with uncomplicated malaria living in the lagoon costal area in southern Benin.

## Methods

### Study site and population

This is a randomized trial conducted at three adjacent peri-urban sites: Ladji, Awansori and Toweta, situated in the coastal lagoon area of Cotonou. *Anopheles gambiae *and *Anopheles melas *are the main malaria vectors [10], and the annual entomologic inoculation rate (AEIR) has been estimated at 58 infective bites per person [10,11]. Prevalence of malaria infection in children between two and nine years of age is high and defines this area as hyper-endemic [12].

The local authorities and the population were informed about the study and its objectives in 2001. A census was carried out in 2002–2003. Houses were numbered and information on the household's residents (name, sex, age, relationship with the household head) collected. A list of six to 59 months old children without any obvious chronic illness, with parents willing to participate to the study, and unlikely to emigrate outside the study area was produced. Five hundred and fifty three children (34% of all children of this age group) were randomly chosen from this list to be part of the cohort under surveillance. Parents or legal guardians were asked before inclusion to sign an informed consent form. Refusal to participate did not affect access to basic health services. Parents/guardians were instructed to attend the nearby health facility, where a physician was always available, whenever their child was sick. They were also asked to administer to the child only drugs given or prescribed by the research physician.

Starting from July 2003 up to January 2005, each child was visited twice a week by the research team. Body temperature was measured routinely during home visits and, if the child was febrile (body temperature ≥ 37.5°C), a blood sample for parasitaemia (thick and thin blood film) and for genotyping at a later stage (filter paper Whatman grade 3) was collected. A similar approach was applied to children attending health facilities within the study area. An additional blood sample for PCV was collected in children with a *Plasmodium falciparum *mono-infection with a density between 1,000–200,000/μL. Children were allocated to one of the study treatment groups, i.e. CQ, SP or SP-AS if they met no exclusion criteria: PCV < 25%, severe malaria [13], danger signs (prostration, inability to drink, recent convulsion, persistent vomiting) or other concomitant illness or underlying disease. Allocation to treatment groups was done according to a predefined randomization list and was concealed (sealed opaque envelopes) until final recruitment of the patient. Treatment was administered according to the body weight at the following doses: CQ: 10 mg/kg/day on days 0 and 1, and 5 mg/kg on day 2; SP: 25 mg/kg of sulphadoxine and 1.25 mg/kg of pyrimethamine as a single dose on day 0; SP as before plus artesunate: 4 mg/kg/day for three days. Treatment was administered directly by the study team and patients were observed for one hour. Treatment was repeated if vomiting occurred: a full dose if within 30 minutes and half dose if between 30 minutes and one hour. If vomiting persisted, the patient was referred to the health facility and rescue treatment was given (full course of quinine, according to Benin National Treatment Guidelines [1]). A case-record form was completed for each patient documenting all symptoms prior to treatment, concomitant illness and drug history. Besides the first three days of treatment (days 0, 1 and 2), children were seen at scheduled visits at days three, seven, 14, 21 and 28. In addition, parents/guardians were encouraged to attend the health facilities whenever the child was sick. At each visit, the clinical history, signs and symptoms, body temperature were recorded and a blood sample was collected for parasitaemia and genotyping at a later stage.

### Laboratory methods

Thick blood films were stained with Giemsa (10% for 10 minutes). Parasite density was determined on a thick blood film according to the number of parasites per 200 white blood cells (WBC), and assuming a total WBC count of 8,000/μl. If gametocytes were seen, the gametocyte count was extended at 1,000 WBC. Microscopy examination was totally blinded to the patient's identity and treatment group. PCV, measured by micro-haematocrit centrifugation, was assessed on days 0, 14 and 28. Genotyping, to distinguish between a new infection and a recrudescence, was done on blood samples collected before treatment and at the time of any recurrent parasitaemia and was performed at the Institute of Tropical Medicine, Antwerp, Belgium. DNA was extracted as described elsewhere. Parasite genotyping was done by nested PCR for variable blocks within the merozoite surface protein 1 and 2 (*msp1*and *msp2*) as described previously [14,15]. A recrudescence was defined by observing at least one common band for both markers between the infections before treatment at day 0 and at the time of recurrent parasitaemia. New infection was defined by completely different bands between the two infections in at least one of the two markers.

### Outcome measurements

Outcomes were defined according to the standard WHO classification [16]. Early treatment failure (ETF) was defined as one of the following: i) danger signs/severe malaria at day 1, 2 or 3 with parasitaemia; ii) parasite density at day 2 greater than at day 0; iii) parasitaemia at day 3 with fever (axillary temperature ≥ 37.5°C); iv) parasite density at day 3 equal or greater than 25% of that at day 0. Late clinical failure (LCF) was defined as danger signs/severe malaria or parasitaemia with fever occurring between day 4 and day 28, without having been previously classified as ETF. Late parasitological failure (LPF) was defined as parasitaemia without fever between day 4 and day 28 and without previously meeting any of the criteria for ETF or LCF. An adequate clinical and parasitological response (ACPR) was defined as the absence of parasitaemia by day 28 without previously meeting any of the criteria for ETF, LCF and LPF. The primary outcomes were day-28 failure rates (uncorrected and PCR corrected). Total treatment failure (TTF) was computed as ETF+LCF+LPF. After day 14 only infections confirmed by PCR as recrudescence were considered as treatment failures. Secondary outcomes included fever, parasite and gametocyte clearance.

### Statistical analysis

Data were analysed in STATA version 8.0 (Stata Corporation, College Station, Texas, US). Descriptive statistics were used to summarize baseline values and demography data.

Analysis of variance (ANOVA) was used for normally distributed continuous data. Data not normally distributed were compared by Wilcoxon Rank sum or Kruskal-Wallis tests. Categorical data were compared by calculating the chi-square value or by Fisher's exact test. Data were analysed by Intention-to-Treat (ITT), Per-Protocol (PP) and Kaplan Meier survival analysis to determine the cure rate (PCR corrected) at day 28 in each treatment group. In the ITT analysis, patients lost to follow up were considered as treatment failures (worst case scenario) and the denominator was the number of patients initially randomized to each group. In the PP analysis, patients lost to follow-up and those excluded after enrolment were excluded from the analysis; only patients with a complete follow-up were included in the denominator. Cure rates, fever and parasite clearance were compared using logistic regression models. Odds ratio and 95% CI were estimated from these models. In the Kaplan Meier survival analysis, each patient contributed to the analysis for the time s/he was followed up. Data were censored for patients who did not complete follow up and for new *P. falciparum *infection when estimating outcomes corrected by PCR. In the survival analysis, cure rates were described by Kaplan Meier estimates and compared between groups with a log-rank test. Pair-wise comparisons of treatment efficacy at day 28 were made with a Cox proportional hazards model. Gametocyte carriage rates were estimated as proportion of patients with gametocytaemia and person-gametocyte-weeks (PGW) were calculated to measure gametocyte carriage and transmission potential. The PGW were calculated as the number of weeks in which blood slides were positive for gametocytes during the two first weeks of follow-up after treatment divided by the number of all follow-up weeks and expressed per 1,000 person-weeks. P < .05 was considered statistically significant.

### Ethical approval

The study was approved by the Minister of Health of Benin, the Ethical Committee of the Faculté des Sciences de la Santé, Cotonou, Benin and the Ethical Committee of the Institute of Tropical Medicine, Antwerp. Informed consent was obtained from parents or guardians.

## Results

**Figure 1:**
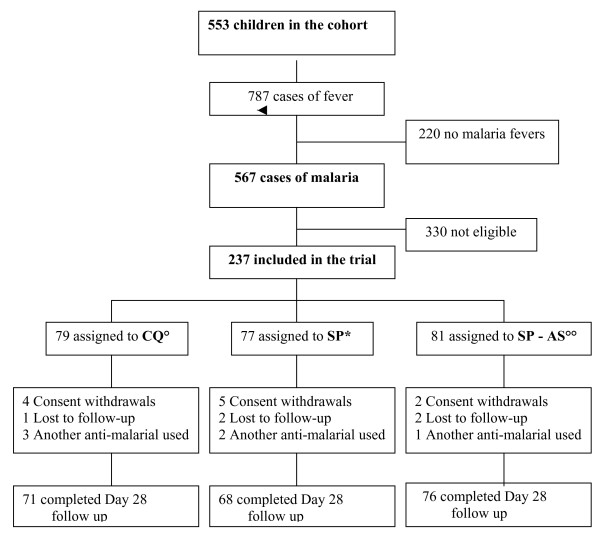
**Trial profile**. ^◦ ^CQ = Chloroquine; *SP = Sulfadoxine-Pyrimethamine; ^◦◦ ^AS = Artesunate.

A total of 553 children, aged between six and 59 months, were included in the study cohort. Among them, between July 2003 and January 2005, 787 fever cases were identified: 567 had a *P. falciparum *infection, of whom 237 fulfilled all inclusion criteria. These children were randomly assigned to one of the study groups: 79 to CQ; 77 to SP and 81 to the combination SP-AS (Figure 1). Most children (90.7%) completed the 28-day follow up and were included in the PP analysis. For the others, 11 parents/guardians withdrew their consent, five children moved out of the study area, and six were excluded because they received another anti-malarial drug. All these children were included in ITT analysis as failures and in the survival analysis as event-free for the time they participated. The three treatment groups were comparable in terms of demographic and clinical characteristics at baseline (Table 1).

**Table 1:** Baseline characteristics by treatment group

	**CQ (n = 79)**	**SP (n = 77)**	**SP-AS (n = 81)**
***Demographic characteristics***			

Sex ratio, Males:Females	36:43	46:31	43:38
Mean age in months [SD]	31.4 [13.8]	35.9 [16.9]	36.9 [16.3]
Mean weight in kg [SD]	13.4 [2.6]	13.7 [3.3]	14.3 [3.0]
***Clinical characteristics***			

Days with fever before diagnosis, median [IQR]*	2 [1–3]	1 [0–3]	2 [1–3]
Mean body temperature, °C [SD]	38.3 [0.8]	38.3 [0.7]	38.2 [0.7]
Bed net use, n (%)	57 (72.1)	63 (81.1)	60 (74.0)
Parasite density (/μl)at Day 0, (geometric mean [range])	16,323 [1,119; 198,107]	20,100 [1,192; 192,398]	20,286 [1,114; 198,609]
Gametocytaemia at day 0, n (%)	6 (7.6)	2 (2.6)	3 (3.7)
Packed cell volume, mean [range]	32.6 [22–43]	34.1 [26–40]	33.5 [23–43]

*IQR = Interquartile range

Fever clearance was significantly more rapid in the SP-AS than in the CQ or SP groups; by day 3 the prevalence of fever was the following: SP-AS: 0/79 (0.0%), SP alone: 4/67 (6.0%), CQ: 6/62 (9.7%) (p = 0.02). Parasite clearance was also faster in the SP-AS than in the other two treatment groups. By day 3, the percentage of children without infection was 94.9% (75/79) in the SP-AS, 56.7% (38/67) in the SP and 56.5% (35/62) in the CQ group (p < 0.001).

In the SP-AS group, no ETF was observed while there were 16 in the SP and 18 in the CQ group (Table 2). By day 14, only one treatment failure (LPF) occurred in the SP-AS group, while there were 8 (two LCF + six LPF) in the SP group, and 21 (six LCF + 15 LPF) in the CQ group. Overall, TTF by day 14 (PP analysis) was 1.3% (1/77) for the SP-AS group, 34.3% (24/70) for SP alone group and 54.9% (39/71) for the CQ group (p < 0.001).

**Table 2:** Clinical and parasitological failure by day 14 and day 28

	**CQ**	**SP**	**SP – AS**
**DAY 14, n**	**71**	**70**	**77**
ACPR	32 (45.1)	46 (65.7)	76 (98.7)
ETF, n (%)	18 (25.4)	16 (22.9)	0 (0.0)
LCF, n (%)	6 (8.5)	2 (2.9)	0 (0.0)
LPF, n (%)	15 (21.1)	6 (8.6)	1 (1.3)
TOTAL FAILURES			
*Per protocol, n (%)*	39 (54.9)	24 (34.3)	1 (1.3)
*Intention to Treat, n (%)*	47/79 (59.5)	31/77 (40.3)	5/81 (6.2)

**DAY 28 (PCR uncorrected), n**	**71**	**68**	**76**

ACPR	18 (25.4)	37 (54.4)	68 (89.5)
ETF, n (%)	18 (25.4)	16 (23.5)	0 (0.0)
LCF, n (%)	8 (11.3)	4 (5.9)	1 (1.3)
LPF, n (%)	27 (38.0)	11 (16.2)	7 (9.2)
TOTAL FAILURES			
*Per protocol, n (%)*	53 (74.6)	31 (45.6)	8 (10.5)
*Intention to Treat, n (%)*	61 (77.2)	40 (51.9)	13 (16.0)

**DAY 28 (PCR corrected)**			

ACPR	20 (28.2)	38 (55.9)	72 (94.7)
ETF, n (%)	18 (25.4)	16 (23.5)	0 (0.0)
LCF, n (%)	8 (11.3)	4 (5.9)	1 (1.3)
LPF, n (%)	25 (35.2)	10 (14.7)	3 (3.9)
TOTAL FAILURES			
*New infections, n (%)*	2 (14.3)	1 (14.3)	4 (57.1)
*Per protocol, n (%)*	51 (71.8)	30 (44.1)	4 (5.3)
*Intention to Treat, n (%)*	59 (74.7)	39 (50.6)	9 (11.1)
*Kaplan Meier survival analysis (%)*	71.8	42.0	5.3

By day 28, seven additional recurrent parasitaemia occurred in the SP-AS group (one LCF and six LPF), seven in the SP group (two LCF and five LPF), and 14 in the CQ group (two LCF and 12 LPF). Thus, the failure rate (PCR uncorrected) was 10.5% (8/76) for the SP-AS, 45.6% (31/68) for the SP alone and 74.6% (53/71) for the CQ group. After PCR correction, these rates were 5.3% (4/76), 44.1% (30/68) and 71.8% (51/71), respectively (Table 2). The odds of failure was significantly higher in the CQ than in the SP alone group (OR = 3.2; 95% CI 1.5; 7.0) (p = 0.002); and for the latter it was significantly higher than in the SP-AS group (OR = 14.2; 95% CI: 4.3; 58.5) (p < 0.001). Compared to SP-AS, the odds of failure for CQ was 46 times higher (OR = 45.9; 95% CI: 14.0; 188.9) (p < 0.0001).

**Figure 2:**
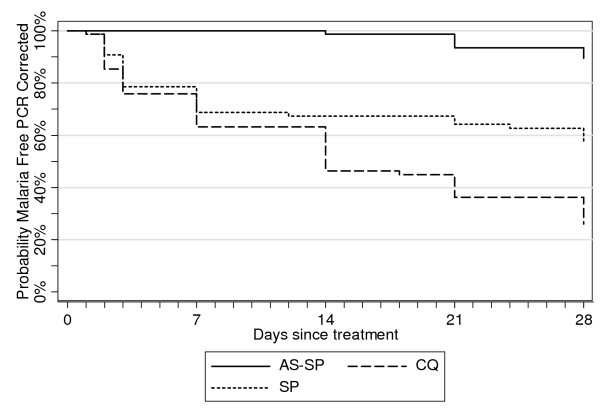
Kaplan Meier survival curves (PCR Adjusted) by treatment group.

Treatment failure rates estimated by Kaplan-Meier survival analysis (PCR-corrected) were similar (SP-AS 5.3%, SP alone 42.0%, CQ 71.8%) to the PP analysis. Differences between study arms were highly significant (Log-Rank p < 0,001) and the hazard of failure estimated by Cox regression was 20 times higher with CQ compared to SP-AS (HR = 20.7, 95% CI: 7.5; 57.5) (p < 0.001). For SP alone versus SP-AS, the assumption of proportional hazards was respected only after day 3: the hazard for treatment failure was almost six times higher with SP compared to SP-AS (HR = 5.7, 95% CI: 1.9; 18.2) (p = 0.002) (Figure 2).

Before treatment, no significant difference in gametocyte prevalence was observed between groups although gametocyte carriages tended to be higher in the CQ group (Table 1). Gametocyte carriage was significantly lower in the SP-AS group compared to the two others, and by day 28 no patient in this group had gametocytes (Table 3). Gametocyte carriage rate expressed as person-gametocyte-weeks (PGW) was significantly lower in the SP-AS group (16.7/1,000) than in the SP group (74.9/1,000) (p = 0.008). There was no difference in PGW when comparing the SP-AS group to the CQ group (p = 0.33).

**Table 3:** Gametocyte carriage by treatment group

	**CQ**	**SP**	**SP-AS**	**p value**
**Gametocyte prevalence, n (%)**				
Day 0	79 (7.6)	77(2.6)	81(3.7)	0.31
Day 7	53 (5.7)	56(16.1)	77(3.9)	0.03
Day 14	45(6.7)	47(10.6)	77(2.6)	0.18
Day 28	25(12.0)	40(10)	72(0.0)	0.02

**Gametocyte incidence, n (%)**				
Day 7	49(6.1)	55(16.4)	75(2.7)	0.01
Day 14	41(4.9)	47(10.6)	75(2.7)	0.17
Day 28	24(12.5)	40(9.8)	70(0.0)	0.02

**Person-gametocyte-weeks **(/1000 person-weeks)	6/154 (39.0)	14/187 (74.9)	5/301 (16.7)	0.02^†^

†Kruskal-Wallis p-value.

All children have been closely followed up by home visits during 28 days (daily the first week, then weekly). In total, 28 serious adverse events were identified (three SP-AS, 15 CQ, 10 SP) (p = 0.15). No death was reported. No severe malaria cases were observed in the SP-AS group while there were two cases of convulsions in the other two groups (one CQ and one SP) and five cases of severe anaemia (three CQ and two SP) (Table 4). Seven patients had serious adverse events clearly related to acute respiratory infection (three SP-AS, two CQ, two SP, p = 0.12) and one patient in the SP group had pyomyositis.

**Table 4:** Serious adverse events by treatment group

	**CQ (N = 79)**	**SP (N = 77)**	**SP – AS (N = 81)**
Patients with any SAE, n (%)	15 (19.0)	10 (13.0)	3 (3.7)^‡^
- Severe anaemia	3	2	0
- Acute respiration infection	2	2	3
- Seizure	1	1	0
- Pyomyositis	0	1	0

(% calculated on the total number of patients). ‡ Comparison using Fisher's Exact test for SP-AS *vs *CQ (p = 0.002) and SP-AS *vs *SP (p = 0.043)

## Discussion

The combination SP-AS was safe, well-tolerated and significantly more efficacious than the two other treatments. Children treated with SP-AS had also a faster fever and parasite clearance. At day 28, the PCR-corrected TTF for the SP-AS group was only 5.3%, an extremely low figure compared to the other two treatments. These findings are comparable to the 28-day PCR-corrected cure rates recently reported from Ghana, (94.5%) [17] and eastern Sudan (97.5%) [18]. The high treatment failure observed in our study for both CQ and SP monotherapies suggests a high prevalence of mutations linked to drug resistance [19,21]. This may compromise the efficacy of ACT in which the partner drug to the artemisinin-derivative shares common resistance markers with either CQ or SP. For example, when considering that there may be cross-resistance between CQ and amodiaquine (AQ) [22-24], the recently available fixed-dose combination AQ-artesunate, one of the treatment included in the national antimalarial drug policy, may be less efficacious than expected. It is often stated that in an ACT the partner drug to the artemisinin derivative should have a good efficacy and this because a 3-day treatment with artesunate is insufficient to clear an infection. If the partner drug is not effective, it is likely that a large number of infections, incompletely treated, could re-appear during the patient follow up. It is, therefore, surprising that in this study, despite the high risk of treatment failure in the children treated with SP alone, SP-AS had such a good efficacy. Even the PCR-uncorrected results are extremely good as only about 10% of the children in the SP-AS group experienced a failure or a recurrent infection, despite the high transmission intensity, as compared to almost half of children treated with SP alone. In Thailand, combining artesunate with mefloquine considerably improved the efficacy of treatment, despite the high resistance to mefloquine itself [25]. However, the intensity of malaria transmission in Benin is much higher than in Thailand and new infections are more likely to occur shortly after treatment. However, a longer follow up may have detected more cases of recurrent parasitaemia or even recrudescence [26]. It is also possible that the high bed net use among the study children may have limited their risk of new infections.

As expected, gametocyte carriage in children treated with SP-AS was much lower than in the other treatment groups. A decrease in malaria transmission after the introduction of ACT has already been reported in Thailand and it has been suggested that this was linked to the lower gametocyte production in patients treated with ACT [25]. However, considering that malaria transmission in Africa, more specifically in the coastal lagoon area of Cotonou, is much higher than in south-east Asia, it is unclear whether the introduction of ACT will have a major impact on transmission. Nevertheless, anecdotic reports of a lower-than-expected number of malaria patients at health facilities in several African countries have been produced [27]. It is unclear whether this is a true phenomenon and, if yes, this is due to the introduction of ACT or also to other interventions such as insecticide-treated bed nets.

## Conclusion

Benin recently chose artemether-lumefantrine and amodiaquine-artesunate as recommended treatments for uncomplicated malaria. This decision was taken in 2004 but it is unfortunate to notice that these ACT are not available yet at peripheral health facilities. Other African countries such as Burkina Faso are in a similar situation, i.e. the national drug policy has been changed but not implemented yet (Tinto Halidou., personal communication). In Benin, considering that SP and AS are easily available and their combination has an acceptable efficacy, SP-AS may be used whenever the recommended standard treatments are hopefully temporarily unavailable. However, because SP is also used in pregnant women as intermittent preventive treatment (IPTp), its combination with AS should not be widely employed in malaria patients as this may compromise the efficacy of IPTp.

## Competing interests

The author(s) declare that they have no competing interests.

## Authors' contributions

**AN**: contributed to the study design, study coordination and supervision, field work, data entry, cleaning and analysis, and paper writing.

**AE**: contributed to the study design, data analysis and review of the paper.

**DG**: contributed to the study design and field work.

**CVO**: contributed to the parasite genotyping.

**CA**: contributed to the planning and implementation of the field work.

**HvL**: contributed to the data management.

**JM**: contributed to the statistical analysis.

**MA**: contributed to the implementation of the study.

**MC**: contributed to the study design and paper reviewing.

**AM**: contributed to the study design, data analysis and interpretation and manuscript review.

**UDA**: contributed to study design, protocol writing, data analysis, interpretation and supervision, and manuscript review.
